# Albumin-induced coagulopathy is less severe and more effectively reversed with fibrinogen concentrate than is synthetic colloid-induced coagulopathy

**DOI:** 10.1186/cc12312

**Published:** 2013-03-19

**Authors:** DW Winstedt, JH Hanna, US Schött

**Affiliations:** 1 Lund University, Lund, Sweden

## Introduction

Volume resuscitation is essential to restore normovolemia during hemorrhagic shock, burns and sepsis. However, synthetic colloids cause dilutional coagulopathy. The aims were to determine whether the natural colloid albumin induces a lesser degree of coagulopathy compared with synthetic colloids, and the comparative effectiveness of fibrinogen concentrate to reverse coagulopathy following dilution with these solutions.

## Methods

Rotational thromboelastometry-based tests were used to examine coagulation parameters in samples from 10 healthy volunteers, in undiluted blood and samples diluted 1:1 with saline, Ringer's acetate, hydroxyethyl starch (HES) 130/0.4, buffered HES 130/0.4, 3% dextran 60, 6% dextran 70 or 5% albumin. Samples were analyzed before and after addition of 2 mg/ml fibrinogen concentrate.

## Results

EXTEM maximum clot firmness (MCF), clot formation time (CFT) and α angle (AA) decreased significantly upon dilution with all colloid solutions (*P <*0.001), although a significantly greater coagulopathic effect was seen for samples diluted with synthetic colloid solutions versus albumin (*P *≤0.001). A significant reduction in the platelet component of clot strength (EXTEM MCF - FIBTEM MCF) was seen for samples diluted with synthetic colloids (*P <*0.001) but not albumin (*P *= 0.10). Following addition of fibrinogen, FIBTEM MCF, EXTEM MCF and EXTEM AA were significantly higher, and EXTEM CFT was significantly shorter in samples diluted with albumin versus those treated with HES or dextran (*P *≤0.001).

## Conclusion

Hemodilution using albumin induced a lesser degree of coagulopathy compared with the synthetic colloids HES and dextran. In addition, albumin-induced coagulopathy was more effectively reversed following addition of fibrinogen concentrate compared with coagulopathy induced by synthetic colloids.

